# Is spontaneous normalization of systolic blood pressure within 24 hours after ischemic stroke onset related with favorable outcomes?

**DOI:** 10.1371/journal.pone.0224293

**Published:** 2019-10-22

**Authors:** Seo Hyun Kim, Ji In Kim, Ji-Yong Lee, Chan Ik Park, Jin Yong Hong, Sung-Soo Lee

**Affiliations:** Department of Neurology, Yonsei University Wonju College of Medicine, Wonju, Gangwon-do, Republic of Korea; Universita degli Studi di Roma La Sapienza, ITALY

## Abstract

**Background:**

In acute ischemic stroke, blood pressure (BP) tends to rise initially and fall to a baseline level within 24–48 hours. Previous studies reported several different effects of BPs during acute ischemic stroke on clinical outcomes, which was partly due to the different time intervals from stroke onset to BP measurement.

**Methods:**

All patients with acute ischemic stroke (onset ≤3 hours) who lived independently before the stroke, were consecutively enrolled for a 62-month period. BPs at 0, 12, and 24 hours after admission were collected. A favorable outcome was defined as a modified Rankin Scale (mRS) score 0–2 at discharge. For different standards of BP management, patients were grouped and analyzed according to intravenous (IV) tissue plasminogen activator (tPA) treatment and favorable outcome.

**Results:**

Among the 446 enrolled patients, 227 patients underwent IV tPA treatment and 216 had mRS score 0–2 at discharge. Patients with favorable outcomes had lower initial NIH Stroke Scale (NIHSS) scores, less frequent progressive neurological deficits, and lower systolic BP (SBP) 12 and 24 hours after admission than patients with unfavorable outcomes, regardless of whether they underwent tPA treatment or not (p <0.05). The BP decreased over a period of 24 hours after admission. In logistic regression analysis, the independent variables associated with favorable outcome were the initial NIHSS score, a progressive neurological deficit, a previous stroke, and the SBP 24 hours after admission in the patients who underwent tPA treatment and the initial NIHSS score and a progressive neurological deficit in the patients who did not undergo tPA treatment (p <0.05).

**Conclusions:**

The SBPs at 12 and 24 hours after admission were lower in acute stroke patients with favorable outcomes than in the other patients, regardless of whether the patients underwent tPA therapy and the SBP at 24 hours was an independent predictor of favorable outcomes among the patients who underwent tPA treatment.

## Introduction

Among the systems that are involved in blood pressure (BP) regulation, the central nervous system and baroreceptor elicit the faster response (within a few seconds) [1]. Most patients with acute ischemic stroke have elevated BPs [2]. Elevated BPs may help the ischemic penumbra to remain viable by enhancing cerebral perfusion and may help patients to recover impaired function, while it may aggravate cerebral edema and hemorrhagic transformation and lead to poor clinical outcomes in acute ischemic stroke patients [2]. Previous studies reported several different effects of BPs during acute ischemic stroke on clinical outcome [3–6], which was partly due to the different time intervals from stroke onset to BP measurement.

The aim of our study was to investigate the relationship between BP and the clinical outcome in acute ischemic stroke patients admitted within three hours after stroke onset.

## Patients and methods

Over sixty two consecutive months patients with acute ischemic stroke who were admitted at the neurology department within three hours after symptom onset, were selected from the Yonsei Wonju Stroke Registry [7], a single hospital-based registry (Wonju Severance Christian Hospital) for acute cerebral ischemia. Patients with pre-stroke functional dependence, the modified Rankin Scale (mRS) score of 3–5, were excluded. Whether or not intravenous (IV) tissue plasminogen activator (tPA) was administered, there are different standards of the upper limit of BP for management: 220/120 mmHg for patients not undergoing tPA treatment; 185/110 for tPA therapy candidates; 185/105 for patients undergoing IV tPA treatment [8]. Therefore, patients were grouped depending on whether or not they had undergone tPA treatment and had favorable outcome. A favorable outcome was defined as mRS score 0–2 at discharge. These groups include (1) patients undergoing tPA treatment with favorable outcomes (TPA FO group); (2) patients undergoing tPA treatment without favorable outcomes (TPA UFO group); (3) patients who are not undergoing tPA treatment and have favorable outcomes (no-TPA FO group); (4) patients who are not undergoing tPA treatment and do not have favorable outcomes (no-TPA UFO group). The inclusion and exclusion criteria for IV tPA treatment were in accordance with the guidelines from the American Heart Association (AHA), except that patients older than 80 years of age were excluded [8]. Antihypertensive drug used prior to stroke was skipped for the first 24 hours and BP was controlled according to the AHA guideline [8]. BP was measured in the arm using an automated noninvasive blood pressure device.

Registry data and medical records were reviewed. The demographics (age, gender, and current smoking status), existing comorbidities (hypertension, diabetes mellitus, hyperlipidemia, atrial fibrillation, and previous stroke), use of antihypertensive agents within 24 hours after admission, initial stroke severity assessed by the NIH Stroke Scale (NIHSS), etiologic subtype classified by the Trial of Org 10172 in Acute Stroke Treatment (TOAST) classification [9], posterior circulation involvement, neurological progression, symptomatic hemorrhagic transformation (HT), and length of stay were compared among the groups. BPs at 0, 12, and 24 hours after admission were collected and BP variation (BPV) for the first 24-hour period was calculated. Posterior circulation stroke was defined as a symptomatic stroke in the territory of the vertebral, cerebellar, or posterior cerebral arteries or the basilar artery [10]. Progressive neurological deficit was defined as worsening of more than 1 point in the NIHSS score during admission. Symptomatic HT was defined as any HT causing progressive neurologic deficit. The use of patient data in this study was approved by the local ethics committee.

### Statistical analysis

The independent t-test was used to compare continuous variables such as age, length of stay, BP, and BPV among the groups. The nonparametric Mann-Whitney U test was performed to compare the NIHSS score among the groups. The chi-square test was also used for analysis of the other categorical variables. Logistic regression analysis was performed to evaluate the independent factors associated with favorable outcome, and odds ratios (ORs) and 95% confidence intervals (CIs) for them were presented. A p value <0.05 was considered significant. All statistical analyses were performed using the SPSS version 24.0 (Armonk, NY: IBM Corp.).

## Results

Four hundred and eighty-two patients with acute ischemic stroke were admitted within 3 hours after stroke onset. After excluding 36 patients who were functionally dependent before stroke onset, 446 patients were enrolled. Of these, 227 patients underwent IV tPA treatment while the remaining 219 patients did not due to: (1) minor or rapidly improving stroke symptoms (n = 175); (2) the observation of a hypodensity greater than one-third of the cerebral hemisphere on brain computed tomography images (n = 17); (3) the ages of patients (more than 80 years; n = 17); (4) a bleeding diathesis (n = 9); (5) a history of intracranial hemorrhage or aneurysm (n = 3); and (6) uncontrolled hypertension (n = 1). Of the patients who underwent IV tPA treatment, 34 were additionally treated using endovascular therapy.

The characteristics of the enrolled patients are shown in Table 1. Favorable outcomes were observed in 76 patients who underwent IV tPA treatment (TPA FO group) and in 140 patients who did not undergo IV tPA treatment (no-TPA FO group). The average length of stay was 14.0 days and patients with favorable outcomes had shorter lengths of stay than those with unfavorable outcomes (10.6±8.3 versus 17.2±15.0 days, p <0.01). Patients with unfavorable outcomes (the TPA UFO and no-TPA UFO groups) had a higher initial NIHSS and more progressive neurological deficits than those with favorable outcomes (the TPA FO and no-TPA FO groups). A history of previous stroke, the use of antihypertensive agents within 24 hours after admission, and symptomatic hemorrhagic transformation were less prevalent in the TPA FO group compared to the TPA UFO group. An advanced age and atrial fibrillation were less common while current smoking and posterior circulation stroke were more prevalent in the no-TPA FO group than in the no-TPA UFO group.

**Table 1:** Patient characteristics.

	Total (n = 446)	TPA			No-TPA		
		FO (n = 76)	UFO (n = 151)	P	FO (n = 140)	UFO (n = 79)	P
Age (years)	68.3 (12.6)	67.4 (10.4)	68.9 (12.2)	0.38	65.1 (12.8)	73.5 (13.6)	<0.01
Female	181 (40.6)	33 (43.4)	61 (40.4)	0.77	54 (38.6)	33 (41.8)	0.75
Current smoker	117 (26.2)	17 (22.4)	38 (26.2)	0.76	48 (34.3)	14 (17.7)	0.01
Hypertension	292 (65.5)	51 (67.1)	98 (64.9)	0.86	86 (61.4)	57 (72.2)	0.15
Diabetes mellitus	98 (22.0)	16 (21.1)	33 (21.9)	1.00	34 (24.3)	15 (19.0)	0.46
Hyperlipidemia	76 (17.0)	16 (21.1)	19 (12.6)	0.14	30 (21.4)	11 (13.9)	0.24
Atrial fibrillation	156 (35.0)	29 (38.2)	64 (42.4)	0.64	33 (23.6)	30 (38.0)	0.04
Previous stroke	77 (17.3)	5 (6.6)	30 (19.9)	0.02	22 (15.7)	20 (25.3)	0.12
Median initial NIHSS (IQR)	6 (3–16)	7 (6–13)	15 (9–21)	<0.01	2 (1–3)	7 (3–18)	<0.01
Initial SBP	158.7 (30.2)	150.8 (29.8)	158.5 (29.1)	0.07	162.0 (30.7)	161.1 (31.1)	0.84
Initial DBP	87.7 (18.6)	88.0 (19.9)	88.1 (18.5)	0.97	89.2 (17.5)	84.0 (19.4)	0.05
SBP 12-hr	141.7 (21.6)	136.2 (21.1)	146.1 (22.8)	<0.01	137.5 (19.3)	146.1 (21.4)	<0.01
DBP 12-hr	79.3 (13.2)	78.7 (14.6)	79.7 (13.2)	0.60	79.3 (12.6)	79.4 (12.8)	0.98
SBP 24-hr	140.1 (22.5)	136.4 (22.2)	145.1 (21.3)	<0.01	135.1 (21.9)	142.9 (24.1)	0.02
DBP 24-hr	78.7 (14.5)	79.0 (16.5)	80.2 (12.8)	0.58	77.1 (14.1)	78.2 (15.9)	0.58
SBPV	18.6 (30.4)	14.4 (30.3)	13.4 (30.4)	0.82	26.8 (30.5)	18.2 (27.6)	0.04
DBPV	9.0 (19.7)	9.0 (19.2)	7.9 (20.5)	0.69	12.1 (19.2)	5.8 (18.9)	0.02
Etiological subtypes (LAA/CE/SA/SOD)		15/31/7/3	44/53/6/6	0.34	24/38/31/5	19/20/7/5	0.08
Posterior circulation stroke	76 (17.0)	7 (9.2)	22 (14.6)	0.35	37 (26.4)	10 (12.7)	0.03
Antihypertensive agents within 24-hr	48 (10.8)	5 (6.6)	33 (21.9)	<0.01	5 (3.6)	5 (6.3)	0.55
Progressive neurological deficit	106 (23.8)	7 (9.2)	65 (43.0)	<0.01	5 (3.6)	29 (36.7)	<0.01
Symptomatic HT	18 (4.0)	0 (0.0)	16 (10.6)	<0.01	1 (0.7)	1 (1.3)	1.00

Values are presented as n (%) or mean (SD), unless otherwise stated. IQR, interquartile range; SBP, systolic blood pressure; DBP, diastolic blood pressure; 12-hr, 12 hours after admission; 24-hr, 24 hours after admission; SBPV, systolic blood pressure variation for the first 24 hours; DBPV, diastolic blood pressure variation for the first 24 hours; NIHSS, National Institute of Health Stroke Scale; HT, hemorrhagic transformation.

**Fig 1:**
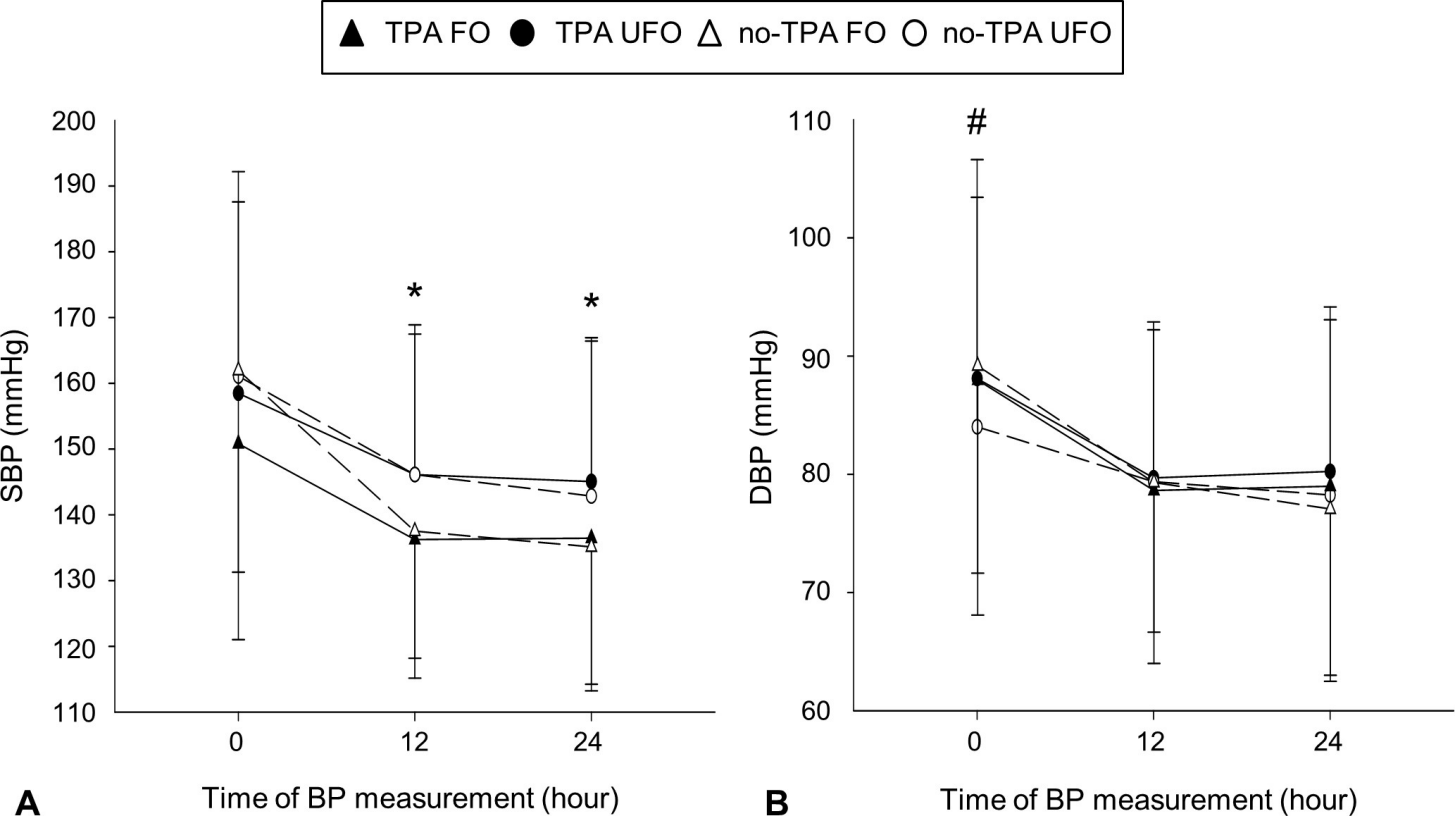
Blood pressure (BP) according to time of BP measurement after admission. (A) Patients with favorable outcomes (TPA FO and no-TPA FO groups) had lower systolic BPs after 12 and 24 hours than those with unfavorable outcomes (TPA UFO and no-TPA UFO groups, *). (B) The initial diastolic BP of patients in the no-TPA FO group was higher than that of patients in the no-TPA UFO group (#).

The mean initial systolic BP (SBP) was 158.7±30.2 mmHg. The mean SBP 12 hours after admission (SBP 12-hr) was 141.7±21.6 mmHg and the mean SBP 24 hours after admission (SBP 24-hr) was 140.1±22.5 mmHg. The mean systolic BPV (SBPV) was 18.6±30.4 mmHg. Additionally, the mean initial diastolic BP (DBP) was 87.7±18.6 mmHg while the mean DBPs 12 hours after admission (DBP 12-hr) and 24 hours after admission (DBP 24-hr) were 79.3±13.2 mmHg and 78.7±14.5 mmHg, respectively. The mean diastolic BPV (DBPV) was 9.0±19.7 mmHg. Patients with favorable outcomes (the TPA FO and no-TPA FO groups) had a lower SBP 12-hr and SBP 24-hr than those with unfavorable outcomes (the TPA UFO and no-TPA UFO groups; Fig 1A). Among 227 patients who underwent tPA therapy, there were 75 patients presenting at the acute stage with large artery occlusion (LAO subgroup) on MR or CT angiography. The patients with favorable outcomes in LAO subgroup tended to have a lower SBP 24-hr (S1 Table). The initial DBPs of the patients in the no-TPA FO group was higher than those of patients in the no-TPA UFO group (Fig 1B). The initial SBPs and the DBPs 12 and 24 hours after admission did not significantly differ between the patients with favorable outcomes and those with unfavorable outcomes (Fig 1). The patients in the no-TPA FO group had higher SBPVs and DBPVs than those in the no-TPA UFO group (Table 1). However, there was no significant difference in the SBPVs and DBPVs between the TPA FO and TPA UFO groups.

Using logistic regression analysis, the independent variables associated with favorable outcomes were a history of previous stroke, SBP 24-hr, initial NIHSS score, and progressive neurological deficits in the patients who underwent IV tPA treatment and initial NIHSS and progressive neurological deficits in the patients who did not undergo IV tPA treatment (Table 2).

**Table 2:** Logistic regression analysis for favorable outcome at discharge.

	TPA		No-TPA	
	OR (95% CI)	p	OR (95% CI)	p
Age	1.01 (0.97–1.04)	0.80	0.97 (0.93–1.01)	0.11
Current smoker	0.54 (0.22–1.33)	0.18	2.25 (0.75–6.80)	0.15
Atrial fibrillation	1.26 (0.58–2.75)	0.56	1.35 (0.47–3.86)	0.58
Previous stroke	0.25 (0.08–0.81)	0.02	0.94 (0.31–2.87)	0.92
SBP 24-hr	0.98 (0.96–1.00)	0.03	0.99 (0.97–1.01)	0.53
SBPV	0.99 (0.97–1.01)	0.19	0.99 (0.97–1.01)	0.42
DBPV	1.01 (0.98–1.03)	0.58	1.00 (0.98–1.03)	0.90
Initial NIHSS	0.81 (0.76–0.87)	<0.01	0.68 (0.57–0.80)	<0.01
Posterior circulation stroke	0.65 (0.18–2.27)	0.50	1.22 (0.43–3.49)	0.71
Progressive neurological deficit	0.10 (0.04–0.26)	<0.01	0.04 (0.01–0.13)	<0.01

SBP 24-hr, systolic blood pressure at 24 hours after admission; SBPV, systolic blood pressure variation for the first 24 hours; DBPV, diastolic blood pressure variation for the first 24 hours; NIHSS, National Institute of Health Stroke Scale.

## Discussion

The BP stabilized over a 24-hour period after admission in all groups. The SBP 12-hr and 24-hr of patients with favorable outcomes were lower than those of patients with unfavorable outcomes regardless of whether they underwent tPA treatment or not. Additionally, the SBP 24-hr of patients who underwent tPA treatment was an independent predictor of favorable outcomes at discharge.

An elevated BP in the acute stage of stroke is common and occurs in about 75% of all cases, which may attribute to: (1) inadequately treated or undetected hypertension; (2) autonomic dysfunction, such as blunted baroreflex and increased sympathetic tone from stoke lesions involving the autonomic nervous system; (3) stress response [2, 11]. The elevated BP decreases over several days [2, 12]. Therefore, the first BP measured tends to be the highest among the measured BPs. Inconsistent results have been reported in studies on the BP during acute ischemic stroke [3–6]. This may be partly because the time interval between the stroke onset and the first BP measurement varies in different studies. This study enrolled homogenous patient group in the time interval from the stoke onset to the initial BP measurement. The time interval was less than 3 hours. In this study, the initial BP tended to be the highest. However, it was not related with a favorable outcome.

Because of different standards of BP management [8], the patients were analyzed separately according to whether or not they underwent recanalization therapy. According to recent recanalization studies [13–15], the SBP of recanalyzed patients falls significantly in the first 24 hours and are correlated with favorable outcomes. Although recanalization after tPA therapy was not evaluated in this study, the lower SBP 12-hr and 24-hr in the TPA FO group may be partly due to recanalization. If a favorable outcome was defined as mRS 0–1 for the patients who underwent IV tPA treatment and did not undergo additional endovascular therapy, the SBP 24-hr of the patients with favorable outcomes were lower than those of patients with unfavorable outcomes (S2 Table).

Patients in the no-TPA FO group had a lower SBP 12-hr and 24-hr as well as those in the TPA FO group. Among the patients who did not undergo tPA therapy, 175 patients (80%) had minor or rapidly improving stroke symptoms and 140 patients (64%) had favorable outcomes. Rapid recanalization of the occluded vessel and functional recovery may explain the rapidly improving stroke symptoms [16, 17] and may also explain the lower SBP 12-hr and 24-hr in the no-TPA FO group.

The SBPV and DBPV in the no-TPA FO group were higher than those in the no-TPA UFO group. This may be associated with the low SBP 12-hr and 24-hr and high initial DBP in the no-TPA FO group. There were conflicting results concerning the BPV and functional outcomes in acute stroke [18]. Unlike the SBP, the correlation between the DBP and the functional outcome after acute stroke was somewhat weak. Isolated high SBPs were also reported during other stressful conditions as well as acute ischemic stroke [19–21]. This suggests the existence of different mechanisms for regulating the SBP and DBP. More patients in the TPA UFO group received antihypertensive agents within 24 hours after admission compared to those in the TPA FO group. This may reflect the unstable state of BP of patients in the TPA UFO group during the 24 hours after admission. A similar result was reported in a recent research [22].

As shown in previous studies [23, 24], the initial NIHSS and a progressive neurological deficit were predictors of favorable outcomes in all enrolled patients at discharge. Many recent stroke trials use 4 point deterioration in NIHSS as definition of progressive stroke and symptomatic hemorrhagic transformation [25]. We chose the 1 point deterioration for more sensitive definition [7, 26, 27]. In patients who underwent tPA therapy, the SBP 24-hr and a history of previous stroke were also predictors of favorable outcomes. Because the present study is an observational study, this result does not imply that lowering the SBP within the first 24 hour will have a beneficial effect on the stroke outcome. There is insufficient evidence that lowering the BP during the acute stage of stroke has a beneficial effect on the outcomes according to Cochrane Database [28].

This study has several limitations. This study was a retrospective study that involved patient data from only one center. We also analyzed BP measurements at arbitrary selected points in time instead of analyzing the BP measurements continuously over the first 24-hour period after admission. We did not evaluate whether recanalization of the occluded vessels was occurred. A lot of patients presenting at the acute stage with large artery occlusion had unfavorable outcomes, which was due to old version protocol for endovascular treatment and outdated devices over a 62-month study period (S1 Table). Additionally, the discharge mRS may be insufficient criterion for longer functional outcome although it was reported to be strongly correlated with the 3-month mRS [29]. Spontaneous decrease of SBP to perinormal level within 24 hours after ischemic stroke onset may be predictor of favorable outcome. Further prospective multicenter studies over shorter periods of time would be needed to verify the assumption and overcome the limitations.

## Supporting information

S1 Table Patient characteristics in LAO subgroup. (DOCX)

S2 Table Patient characteristics in TPA group. (DOCX)
