# Supplementary material for: Is spontaneous normalization of systolic blood pressure within 24 hours after ischemic stroke onset related with favorable outcomes?
Source: PLoS One. 2019 Oct 22;14(10):e0224293. doi: 10.1371/journal.pone.0224293 (PMC6804986; doi:10.1371/journal.pone.0224293)
Supplement: S1 Table — (DOCX) [file pone.0224293.s001.docx]

**S1 Table. Patient characteristics in LAO subgroup.**

|  | LAO subgroup | | |
| --- | --- | --- | --- |
|  | FO  (n = 10) | UFO  (n = 65) | p |
| Age (years) | 67.2 (11.6) | 68.3 (12.9) | 0.80 |
| Female | 4 (40.0) | 30 (46.2) | 0.98 |
| Current smoker | 1 (10.0) | 15 (23.1) | 0.60 |
| Hypertension | 8 (80.0) | 42 (64.6) | 0.55 |
| Diabetes mellitus | 3 (30.0) | 15 (23.1) | 0.94 |
| Hyperlipidemia | 2 (20.0) | 6 (9.2) | 0.63 |
| Atrial fibrillation | 5 (50.0) | 28 (43.1) | 0.95 |
| Previous stroke | 0 (0.0) | 8 (12.3) | 0.53 |
| Median initial NIHSS (IQR) | 15 (9-19) | 17 (11-21) | 0.77 |
| Initial SBP | 147.8 (18.5) | 156.7 (27.1) | 0.32 |
| Initial DBP | 93.5 (16.0) | 86.0 (17.8) | 0.21 |
| SBP 12-hr | 144.3 (24.5) | 144.0 (25.6) | 0.98 |
| DBP 12-hr | 80.4 (15.6) | 77.6 (12.9) | 0.54 |
| SBP 24-hr | 129.1 (18.2) | 141.7 (22.3) | 0.09 |
| DBP 24-hr | 78.1 (13.1) | 77.8 (14.0) | 0.95 |
| SBPV | 18.6 (30.2) | 14.9 (28.8) | 0.71 |
| DBPV | 15.4 (23.0) | 8.2 (22.3) | 0.35 |
| Posterior circulation stroke | 0 (0.0) | 5 (7.7) | 0.82 |
| Antihypertensive agents within 24-hr | 0 (0.0) | 13 (20.0) | 0.27 |
| Progressive neurological deficit | 1 (10.0) | 31 (47.7) | 0.06 |
| Symptomatic HT | 0 (0.0) | 9 (13.8) | 0.46 |

Values are presented as n (%) or mean (SD), unless otherwise stated. IQR, interquartile range; SBP, systolic blood pressure; DBP, diastolic blood pressure; 12-hr, 12 hours after admission; 24-hr, 24 hours after admission; SBPV, systolic blood pressure variation for the first 24 hours; DBPV, diastolic blood pressure variation for the first 24 hours; NIHSS, National Institute of Health Stroke Scale; HT, hemorrhagic transformation.
