# Supplementary material for: Is spontaneous normalization of systolic blood pressure within 24 hours after ischemic stroke onset related with favorable outcomes?
Source: PLoS One. 2019 Oct 22;14(10):e0224293. doi: 10.1371/journal.pone.0224293 (PMC6804986; doi:10.1371/journal.pone.0224293)
Supplement: S2 Table — (DOCX) [file pone.0224293.s002.docx]

**S2 Table. Patient characteristics in TPA group.**

|  | IV TPA Only | | | IV TPA and ET | | |
| --- | --- | --- | --- | --- | --- | --- |
|  | FO  (n = 29) | UFO  (n = 164) | P | FO  (n = 6) | UFO  (n = 28) | p |
| Age (years) | 66.7 (11.3) | 69.0 (11.4) | 0.30 | 65.5 (12.9) | 66.9 (13.3) | 0.81 |
| Female | 11 (37.9) | 70 (42.7) | 0.78 | 3 (50.0) | 10 (35.7) | 0.85 |
| Current smoker | 11 (37.9) | 38 (23.2) | 0.15 | 0 (0.0) | 6 (21.4) | 0.51 |
| Hypertension | 22 (75.9) | 106 (64.6) | 0.33 | 4 (66.7) | 17 (60.7) | 1.00 |
| Diabetes mellitus | 7 (24.1) | 34 (20.7) | 0.87 | 2 (33.3) | 6 (21.4) | 0.93 |
| Hyperlipidemia | 6 (20.7) | 27 (16.5) | 0.77 | 1 (16.7) | 1 (3.6) | 0.78 |
| Atrial fibrillation | 7 (24.1) | 71 (43.3) | 0.08 | 3 (50.0) | 12 (42.9) | 1.00 |
| Previous stroke | 3 (10.3) | 28 (17.1) | 0.53 | 0 (0.0) | 4 (14.3) | 0.77 |
| Median initial NIHSS (IQR) | 6 (5-9) | 12 (7-19) | <0.01 | 17 (12-20) | 15 (11-20) | 0.89 |
| Initial SBP | 149.4 (27.0) | 157.2 (31.1) | 0.21 | 147.2 (13.6) | 157.1 (24.5) | 0.35 |
| Initial DBP | 86.7 (16.2) | 88.7 (19.7) | 0.60 | 93.5 (21.0) | 84.6 (17.1) | 0.28 |
| SBP 12-hr | 136.6 (20.6) | 144.0 (21.2) | 0.08 | 144.7 (26.4) | 141.9 (31.3) | 0.84 |
| DBP 12-hr | 77.5 (14.3) | 80.4 (13.1) | 0.28 | 80.0 (19.3) | 74.9 (14.9) | 0.48 |
| SBP 24-hr | 135.1 (21.9) | 144.7 (22.0) | 0.03 | 126.0 (18.6) | 138.3 (19.7) | 0.17 |
| DBP 24-hr | 77.6 (16.3) | 81.5 (13.5) | 0.16 | 74.5 (13.4) | 73.3 (14.1) | 0.85 |
| SBPV | 14.3 (26.9) | 12.5 (31.3) | 0.77 | 21.2 (25.1) | 18.9 (28.9) | 0.86 |
| DBPV | 9.1 (10.9) | 7.2 (20.7) | 0.46 | 19.0 (29.3) | 11.3 (21.1) | 0.46 |
| Posterior circulation stroke | 3 (10.3) | 22 (13.4) | 0.88 | 0 (0.0) | 4 (14.3) | 0.77 |
| Antihypertensive agents within 24-hr | 2 (6.9) | 29 (17.7) | 0.24 | 0 (0.0) | 7 (25.0) | 0.41 |
| Progressive neurological deficit | 2 (6.9) | 57 (34.8) | <0.01 | 0 (0.0) | 13 (46.4) | 0.10 |
| Symptomatic HT | 0 (0.0) | 10 (6.1) | 0.36 | 0 (0.0) | 6 (21.4) | 0.51 |

Values are presented as n (%) or mean (SD), unless otherwise stated. ERT, endovascular recanalization therapy.
